# Waiting time for radiotherapy in women with cervical cancer

**DOI:** 10.1590/S0034-8910.2015049005953

**Published:** 2016-01-12

**Authors:** Maria Isabel do Nascimento, Gulnar Azevedo e Silva

**Affiliations:** I Departamento de Epidemiologia e Bioestatística. Instituto de Saúde Coletiva. Universidade Federal Fluminense. Niterói, RJ, Brasil; II Departamento de Epidemiologia. Instituto de Medicina Social. Universidade do Estado do Rio de Janeiro. Rio de Janeiro, RJ, Brasil

**Keywords:** Uterine Cervical Neoplasms, radiotherapy, Appointments and Schedules, Waiting Lists, Referral and Consultation, Health Services Accessibility, Equity in Access

## Abstract

**OBJECTIVE:**

To describe the waiting time for radiotherapy for patients with cervical cancer.

**METHODS:**

This descriptive study was conducted with 342 cervical cancer cases that were referred to primary radiotherapy, in the Baixada Fluminense region, RJ, Southeastern Brazil, from October 1995 to August 2010. The waiting time was calculated using the recommended 60-day deadline as a parameter to obtaining the first cancer treatment and considering the date at which the diagnosis was confirmed, the date of first oncological consultation and date when the radiotherapy began. Median and proportional comparisons were made using the Kruskal Wallis and Chi-square tests.

**RESULTS:**

Most of the women (72.2%) began their radiotherapy within 60 days from the diagnostic confirmation date. The median of this total waiting time was 41 days. This median worsened over the time period, going from 11 days (1995-1996) to 64 days (2009-2010). The median interval between the diagnostic confirmation and the first oncological consultation was 33 days, and between the first oncological consultation and the first radiotherapy session was four days. The median waiting time differed significantly (p = 0.003) according to different stages of the tumor, reaching 56 days, 35 days and 30 days for women whose cancers were classified up to IIA; from IIB to IIIB, and IVA-IVB, respectively.

**CONCLUSIONS:**

Despite most of the women having had access to radiotherapy within the recommended 60 days, the implementation of procedures to define the stage of the tumor and to reestablish clinical conditions took a large part of this time, showing that at least one of these intervals needs to be improved. Even though the waiting times were ideal for all patients, the most advanced cases were quickly treated, which suggests that access to radiotherapy by women with cervical cancer has been reached with equity.

## INTRODUCTION

Radiotherapy is used to cure and provide palliative treatment for almost all types of cancer. Around 58.0% of all cervical-type cancer cases receive this treatment.^8^ The time spent waiting to begin radiotherapy is considered to be an indicator for quality of care^a^ and seems to have an influence on cancer treatment results.^16^ An audit performed by the *Tribunal de Contas da União* (Federal Court of Accounts) in Brazil showed that 65.9% of the demand for radiotherapy, in 2010, was met and that the average waiting time for the beginning the first session of treatment was 113.4 days.^b^

The International Agency for Research on Cancer (IARC) estimated 14 million new cases of cancer around the world in 2012.^c^ The estimated number of new cancer cases in Brazil was nearly 577 thousand in 2014-2015, with approximately 15 thousand with topography in the cervix.^d^ According to the IARC, an increased number of cervical cancer cases is expected in the coming decades, with 22,211 cases being predicted for 2020 and 27,099 for 2030.

In Brazil, 45.5% of cervical cancer cases diagnosed between 1995 and 2002 were in their advanced stages,^14^ which increased the demand for oncological services that offer radiotherapy and requires an improved resolution rate of these services, since these services deal with cases in which radiotherapy is the option to provide a cure to the disease. For every thousand new cases of cancer, it is estimated that 500 to 600 require oncological surgery, 700 chemotherapy and 600 radiotherapy as part of their program of therapy.^e^ The demand coming from cervical cancer sufferers can be greater, since radiotherapy was recommended for 69.8% of patients with the disease at stages II and III by doctors at the *Instituto Nacional de Câncer *(INCA – National Cancer Institute), between 1999 and 2004.^6^

Improving access to cancer treatment is a matter of great concern in Brazil, which is evidenced by targeted measures for organizing the network of services and providing greater technological resources. In 2012, a law that regulates an acceptable waiting time of up to 60 days for beginning the treatment of new cases of cancer was passed.^f^ Diagnosing the situation, to understand the reasons behind delays, and improved planning for approaching the disease is equally as important as this legislation. It is worth knowing: (i) the total waiting time and the component time intervals imposed on women up to when they begin radiotherapy for cervical cancer; (ii) if any component has a greater impact on the expected waiting time before beginning radiotherapy; and (iii) if the waiting time for tumors referred to potentially curative and palliative radiotherapy is the same.

The objective of this study was to describe the radiotherapy waiting time for cervical cancer sufferers.

## METHODS

This descriptive study presents data from a cohort of women with cervical cancer, who were identified between October 1995 and August 2010 from anatomical pathology or cervical pathology records at a university hospital in Brazil (Nova Iguaçu General Hospital – HGNI). The HGNI is integrated into the Brazilian Unified Health System (SUS), and is located in the Baixada Fluminense region, which is within the Metropolitan Region of Rio de Janeiro State, Brazil. This hospital is home to a colposcopy unit which is designed for clarifying and treating lesions, which are the precursor to cancer of the lower genital tract, as well as for diagnosing cervical cancer. After initial diagnosis and care, patients with the invasive cervical disease are forwarded for treatment at specialized public units in the city of Rio de Janeiro or to the *Serviço Isolado* (Specialized Isolated Service) – a specialized unit, accredited by SUS, for Radiotherapy and Chemotherapy, in the Baixada Fluminense region.

The 667 new cases of cervical cancer with no treatment were identified upon diagnosis at the HGNI during the period. 51 of these individuals had died before starting cancer treatment, 109 were selected for primary surgical treatment and 26 did not have sufficient information to allow the following identification. Thus, 481 patients who had been recommended for exclusive radiotherapy were eligible for the study. 128 of these cases with treatment only at oncology units in the capital were excluded, where a much more comprehensive network of establishments enabled by the SUS for radiotherapy exists,^g^ compared to the single specialized unit operating in the Baixada Fluminense region. Of the 353 remaining cases, eight were excluded for refusing the recommended treatment and three, despite having been referred, for having their radiotherapy contraindicated. The study population was made up of 342 women who had been subjected to radiotherapy at the Specialized Unit of Radiotherapy and Chemotherapy located in the Baixada Fluminense.

The average age of these patients was 51.7 years old (SD: 13.8 years; variation: 25 to 90 years). The morphological analysis showed a higher frequency of squamous tumors (90.9%), followed by adenocarcinoma (5.8%), adenosquamous (1.5%) and other types (1.8%). The clinical staging showed that the disease had spread further than the cervical structure in most cases (90.4%).

The analyzed information were obtained from the records of patients who had received care at the HGNI and at the Specialized Unit as well as from computer files at the Cancer Hospital Registry at the Specialized Unit. The mortality database^h^ from the Mortality Information System for the State of Rio de Janeiro was also consulted for complementary information. Data were collected about demographic factors, relating to the tumor, on referral, and treatment conditions. The most extensive data analyzed were the lengths of total waiting time and their component intervals, whose calculations were made as follows:

- Interval between the diagnostic confirmation and the beginning of radiotherapy (total waiting time): time (in days) elapsed between registration of the histopathological diagnosis in the patient’s medical chart at the HGNI and the first session of radiotherapy at the Specialized Unit.
- Interval between the diagnostic confirmation and the first oncological consultation at the Specialized Unit: time (in days) elapsed between registration of the histopathological diagnosis in the patient’s medical chart at the HGNI and the first oncological consultation at the Specialized Unit.
- Interval between the first oncological consultation at the Specialized Unit and the first radiotherapy session: time (in days) elapsed between the first oncological consultation at the Specialized Unit and the first radiotherapy session.

Analyzing the total waiting time was done according to the following categories: ≤ 15 days *versus* > 15 days; ≤ 30 days *versus* 30 days >; ≤ 60 days *versus* > 60 days; ≤ 90 days *versus* > 90 days; ≤ 120 days *versus* > 120 days.

The other analyzed variables were: age at diagnosis (**≥** 50 years *versus* < 50 years), clinical staging (tumors up to IIA; tumors IIB-IIIB; tumors IVA-IVB), established according to criteria set out by the International Federation of Gynecology and Obstetrics (FIGO).^2^

The analysis included estimating the mean, standard deviation (SD), median, interquartile range (IQR), absolute and relative frequencies. Nonparametric tests were applied to compare the continuous variables (Kruskal Wallis test) and categorical variables (Chi-square test), considering a 5% significance level.

This study was approved by the Committee of Ethics in Research at the HGNI (Process 259,780, March 26, 2013).

## RESULTS

All 342 women received treatment by external specialized radiotherapy or combined with complementary therapies, at the Specialized Unit of Radiotherapy and Chemotherapy, which resulted in eight different treatment schemes (Table 1).

**Table 1:** Waiting Time for radiotherapy in cervical cancer: distribution of selected characteristics of the 342 cases treated. Baixada Fluminense, RJ, Southeastern Brazil, 1995 to 2010.

Variable	n	%
Age group (years)		
20 to 29	14	4.1
30 to 39	51	14.9
40 to 49	100	29.2
50 to 59	87	25.4
60 to 69	45	13.2
70 to 79	36	10.5
80 and over	9	2.6
Clinical staging		
IA to IB	33	9.6
IIA to IIB	135	39.5
IIIA to IIIB	157	45.9
IVA to IVB	17	5.0
Morphology		
Squamous	311	90.9
Adenocarcinoma	20	5.8
Adenosquamous	5	1.5
Other	6	1.8
Treatment scheme with External Radiotherapy +		
Brachytherapy and chemotherapy	60	17.5
Brachytherapy	94	27.5
Reinforcement	20	5.8
Chemotherapy	58	17.0
Chemotherapy and reinforcement	31	9.1
Brachytherapy and reinforcement	1	0.3
Brachytherapy and reinforcement and chemotherapy	1	0.3
External specialized radiotherapy	77	22.5

Waiting time for radiotherapy, which was evaluated using the median of the interval between the diagnostic confirmation and the first radiotherapy session (total waiting time) over the period (1995 to 2010), was 41 days (IQR: 50 days). The variation was almost six fold, from 11 days in the initial biennium (1995 to 1996) to 64 days in the final biennium (2009 to 2010). The median of the interval between the diagnostic confirmation and the first oncological consultation at the Specialized Unit and between the first oncological consultation and the first radiotherapy session was 33 days (IQR: 50 days) and four days (IQR: seven days), respectively. With the exception of the 1995-1996 biennium, for the interval between the first oncological consultation and beginning radiotherapy, the extension of the averages of the analyzed time components was systematically greater than the extension of the medians, over all the periods. This finding pointed to the presence of extreme values on the right, which represents cases that, despite having had access to radiotherapy, were subject to long delays in some cases (Table 2).

**Table 2:** Waiting Time for radiotherapy in cervical cancer: average, median and interquartile range counted in total waiting day and total interval components by biennia. Baixada Fluminense, RJ, Southeastern Brazil, 1995 to 2010.

Biennium	Total interval between diagnostic confirmation and beginning radiotherapy at the specialized unit			Interval between diagnostic confirmation and first oncological consultation at the specialized unit			Interval between first oncological consultation and beginning radiotherapy at the specialized unit		

	Mean	Median	IQR*	Mean	Median	IQR	Mean	Median	IQR
1995 to 1996	21.6	11	12	17.1	5	2	4.5	5	5
1996 to 1997	15.1	11	15	8.5	6	6	6.5	3	5
1999 to 2000	35.3	15	40	30.6	12	35	4.6	1	1
2001 to 2002	29.9	17	26	21.5	7	20	8.3	6	8
2003 to 2004	40.6	30	39.5	30.1	16.5	38	10.4	5	9.5
2005 to 2006	77.1	58	39	73.2	56.5	35	3.9	1	5.5
2007 to 2008	69.1	60	37	62.9	56	31	6.2	4	7
2009 to 2010	82.1	64	26	71.2	53	22	10.9	8.5	8
1995 to 2010	48.3	41	50	41.1	33	50	7.2	4	7

* IQR (interquartile range).

The clinical staging analysis showed that 16.1% of women were in the earlier stages (up to IIA); 78.9% were classified in the intermediate stages (IIB-IIIB) and 5.0% had the disease at more advanced stages (stages IVA-IVB). These conditions were shown to have a statistically significant relationship (p = 0.003) with the total waiting time, with medians of 56 days, 35 days and 30 days for cases classified within groups up to IIA, from IIB to IIIB, and IVA-IVB, respectively.

The schematic representation of the different components of the waiting time showed that the increase in the interval between the diagnostic confirmation and the radiation first session was slighter between the biennia 1997 to 1998 and 1999 to 2000, which then deteriorated more sharply from 2001 and 2002. Throughout the period, the interval between the first oncological consultation and beginning radiotherapy remained constant, it did not exceed 10 days (Figure).

**Figure:**
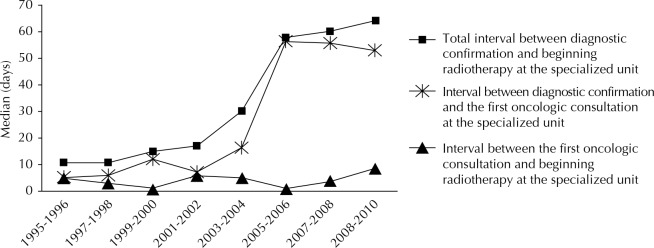
Waiting time for radiotherapy on the cervical cancer: evaluation of the median for total waiting time and components intervals over the biennia included in the study. Baixada Fluminense, RJ, Southeastern Brazil, 1995 to 2010.

In regards to the total waiting time, the proportion of cases with access to treatment was 30.4%, 43.8%, 72.2%, 88.3% and 94.1% within up to 15 days, 30 days, 60 days, 90 days and up to 120 days, respectively. At the end of the first three biennia, over half of the cases began their radiotherapy within 15 days. This proportion fell from 44.9% (2001 to 2002) to zero (2005 to 2006 and 2009 to 2010) (Table 3).

**Table 3:** Total waiting time from the diagnostic confirmation to beginning radiotherapy on the cervical cancer: proportion of cases with access to treatment within 15 days, 30 days, 60 days, 90 days and 120 days. Baixada Fluminense, RJ, Southeastern Brazil, 1995 to 2010. (N = 342)

Variable	Total		Access within 15 days		Access within 30 days		Access within 60 days		Access within 90 days		Access within 120 days	

	n	%	n	%	n	%	n	%	n	%	n	%
Total			104	30.4	150	43.8	247	72.2	302	88.3	322	94.1
Biennium												
1995 to 1996	10	2.9	7	70.0	9	90.0	9	90.0	9	90.0	10	100
1997 to 1998	47	13.7	34	72.3	41	87.2	46	97.9	47	100	47	100
1999 to 2000	43	12.6	22	51.2	29	67.4	34	79.1	37	86.0	41	95.4
2001 to 2002	49	14.3	22	44.9	34	69.4	43	87.8	47	96.0	47	96.0
2003 to 2004	56	16.4	17	16.3	29	51.8	48	85.7	50	89.3	53	94.6
2005 to 2006	48	14.0	0	0	3	6.2	26	54.2	39	81.2	43	89.6
2007 to 2008	51	14.9	2	1.9	5	9.8	26	51.0	41	80.4	47	92.2
2009 to 2010	38	11.1	0	0	0	0	15	39.5	32	84.2	34	89.5
p				< 0.001		< 0.001		< 0.001		0.032		0.364
Staging												
From IA to IIA	55	16.1	10	18.2	15	27.3	35	63.6	43	78.2	47	85.5
From IIB to IIIB	270	78.9	87	32.2	126	46.7	201	74.4	244	90.4	260	96.3
Of IVA and IVB	17	5.0	7	41.2	9	52.9	11	64.7	15	88.2	15	88.2
p				0.073		0.023		0.206		0.037		0.004
Age group (years)												
≥ 50	177	51.7	57	32.2	82	46.3	128	72.3	155	87.6	165	93.2
< 50	165	48.3	47	28.4	68	41.2	119	72.1	147	90.0	157	95.1
p				0.455		0.341		0.968		0.662		0.447

Access to radiotherapy within 60 days was achieved by a relatively high proportion of women (72.2%), considering the entire period. The proportion reached 90.0% in the first two biennia. However, access to radiotherapy up to 60 days gradually decreased from 2001 to 2002 (87.3%) up to 2009 to 2010, while 39.5% of patients with cervical cancer started radiotherapy, within the aforementioned time limit.

Regardless of the cut-off points used for defining the time intervals (15, 30, 60, 90 and 120 days), the proportion of women with the disease at stages IIB-IIIB and IVA-IVB, who receive radiotherapy more swiftly, was greater than those classified as being in the earlier stages, namely up to IIA.

Access to radiotherapy for women younger than 50 years of age was similar for those aged 50 years or more, which was true for all of the analyzed waiting time cutoff points.

## DISCUSSION

This study provides unprecedented information regarding the time that women diagnosed with cervical cancer at this university hospital waits before starting radiotherapy at the Specialized Unit. Most (72.2%) of the women with the disease were given access to radiotherapy within 60 days after their diagnostic confirmation, according to information recorded in the patient chart. Despite the median waiting time being 41 days, their component intervals influenced differently the time spent waiting for radiotherapy differently. While the interval between the diagnostic confirmation and the first oncological consultation at the Specialized Isolated Service increased from five to 53 days from the first biennium (1995 to 1996) to the last biennium (2009 to 2010), the interval between the first oncological consultation and effective administration of oncologic treatment remained stable, being less than 10 days throughout the period.

Studies on cancer treatment waiting times for are not common around the world, the fact that they differ on the definition for intervals used and types of tumors and therapy administered makes comparing them difficult. One of the few studies on the subject to have been developed in Brazil had the date of the first consultation at the Department of head and neck surgery at the Hospital of Heliopolis, Sao Paulo, SP, Southeastern Brazil, as an initial reference.^1^ The evaluation involved 217 patients who had laryngeal cancer that were diagnosed between 1996 and 2004, the median time for beginning radiotherapy was 58 days.

In Ontario, Canada, the median was of 5.7 weeks from surgery or biopsy to the first radiotherapy session for cervical cancer diagnosed in 2001 and 2002.^3^ From 2011, the Canadian Institute for Health Information established 28 days as the standard time limit for treating cancer with radiotherapy.^i^ This definition considers the waiting time as being the number of days since the patient is judged ready for treatment and the date of the first radiotherapy session. This institution noted that the time spent involved in the oncological therapeutic plan is part of the waiting time, but it did not include the time required for clinical staging of the tumor. More recently (2003 to 2007), cases of the same type, which were diagnosed in Auckland, New Zealand, had a median of 26 days counting from the time of the decision to treat the disease up to the ionizing therapy being administered.^5^

In the United Kingdom, studies that followed the introduction of the ‘NHS Cancer Plan’^a^ provided information regarding the waiting time for radiotherapy and indicated possible causes related to the prolonged intervals. Based on data regarding 7,051 cases of uterine cancer, for which the authors did not specify the location, the median time for beginning radiotherapy was 68 days after the diagnostic confirmation, while 42.3% of cases received their first radiotherapy session within 60 days.^12^ Included within the explanatory factors for waiting time to treat any type of cancer, the authors suggest there were not enough trained staff and equipment, as well as mentioning changes in the management of malignant diseases that lead to a greater demand for radiotherapy and increased workload on staff, which is due to the combined increasing frequency of all cancers, despite the confirmed reduction in new cases of cervical cancer.^15^ Coles et al^7^ evaluated the interval between the first oncological consultation and first radiotherapy session at Addenbrooke’s Hospital, Cambridge, UK, in a group of patients with cervical cancer. The authors pointed out an increase in the median from 14 days (1996) to 18 days (1998) and then to 35 days (2001). In part, the option for more more complex fractionated radiotherapy regimens, accompanied by lack of linear accelerators and trained staff, can explain such findings.

Thus, by critically analyzing this situation it is possible to help planning actions designed to shorten waiting times for cancer treatments. These results indicate that the interval between the diagnostic confirmation and the first oncological consultation was the longest before radiotherapy. This interval involves the implementation of measures with varying degrees of complexity that are not only for establishing the stage of the tumor, but mainly for keeping patients alive who arrive in poor medical conditions when diagnosed.

The line of care for cervical cancer involves numerous services and depends on the availability of different technologies,^4^ which are essential for defining the stage and the clinical recovery of patients. During this phase, highly complex procedures such as blood transfusions, dialysis and medium- and large-scale surgery, which is still difficult to access in some regions of Brazil, are often required. Multi-professional teams are therefore trained to deal with cancer and logistical issues that seem simple, such as the availability of transport, but which are not always easily accessible by patients and health professionals and also contribute to increased waiting time.

Since 2012, the problem of time spent waiting to begin cancer treatment has understandably been given special attention by government authorities in Brazil. Following the passing of the law^f^ that refers to the first stage of treatment received by cancer patients, there was an Ordinance^j^ which provides for the application of the law, which stipulates a maximum of 60 days waiting time before effectively beginning the treatment of malignant neoplasms in the SUS. This puts the issue onto the judicial side, and not as a problem that is scientifically dealt with in the first world. The ‘European Cancer Health Indicator Project’ (EUROCHIP)^9^ aims to analyze cancer inequalities. These inequalities are based on data from population-based cancer records and on indicators of staging, delayed treatment and adherence to clinical protocols. This project has defined that delayed cancer treatment would be an important indicator related to any health system that is being analyzed. The evaluation performed by EUROCHIP 3^13^ concluded that 37.0% of 86 population-based cancer registries in Europe possess data regarding treatment delays, which suggests that this critical issue deserves more attention from all sectors involved. The authors highlighted the importance of the situation analysis as a way to improve understanding as to why treatment was delayed. For these authors, delays can occur not only as a result of the schedule for surgery, chemotherapy and radiotherapy, but mainly due to a lack of suitable available resources, patient education, referral patterns and long waiting periods before receiving expert consultation as well as delays in imaging examinations that are essential to the diagnosis.

Almost 86.0% of cases in this study were at stages II or III upon diagnosis, which is a higher proportion than what was reported during analysis conducted in the cities of Vitoria^10^ (75.4%) and Rio de Janeiro^6^ (68.3%). According to EUROCHIP 3,^13^ the stage at diagnosis represents the delay that patients face to obtain a confirmation for cancer being diagnosed. For these authors, the stage at diagnosis reflects factors such as the population’s understanding of the signs of cancer, access to hospitals and diagnostic centers and the ability of professionals to deal with cancer. Cervical cancer is a type of cancer that is considered totally preventable, but it is still detected at its very advanced stage, therefore publishing information is instrumental in educating the population about the signs and symptoms of the disease, the availability of professionals, examinations at basic health care units and the importance of adhering to screening can optimize resources and detect more early-stage cases. This can in turn reduce the demand for radiotherapy. In addition, some of the women who arrived with an advanced stage of cancer had not undergone any screening, which suggests that the screening programs were not effective in finding all the women from the target age groups in some areas.

The issue raised in this study was to investigate if the waiting time for treating tumors, referred to potentially curative radiotherapy and for palliative radiotherapy, were be the same. While patients who have an earlier-stage of the disease, classified up to IIA, began treatment with a median of 56 days, those at more advanced stages received treatment with a median of 35 days (IIB to IIIB) or a median of 30 days (IVA-IVB). During a population-based study performed in Poland, which was restricted to patients with breast cancer, found an greater average waiting time to begin treatment in cases of localized disease.^11^ The authors argued that diagnosing more advanced stages of the disease is faster because it requires a shorter series of procedures, which are cheaper and easier to implement and interpret, compared with tumors at earlier stages. The finding of this current study suggest that for cervical cancer, the length of waiting time was probably influenced by clinical decisions that were taken to expedite the treatment of more serious cases.

This study has some limitations. It was developed with retrospective data, which were recorded with a purpose that was basically administrative and supportive in nature. However, this study carefully collected the data with relentless adherence to the timeframes, which helped minimize the possible inconsistencies. Despite the waiting time for beginning radiotherapy being an important indicator for cancer treatment, this study remained quantitative in nature and did not evaluate the technical conditions for implementing radiotherapy or the comprehensiveness of the programmed regime. The significant number of cases who had only undergone treatment in oncology units in the capital led to the exclusion of more than 1/4 of patients who had been recommended to receive radiotherapy. Although the impact of such a decision is undeniable, the results are in line the purposes of the study and express an indicator for cancer care that is offered in the Baixada Fluminense region, which has a single unit that is enabled by the SUS for radiotherapy. Faced with the current situation that sees there being great interest on the part of governmental authorities regarding cancer, evaluation of implementation of brachytherapy, which was not done here, can bring about important contributions on the approach to cervical cancer, especially if the interval from the end of the external irradiation is duly taken into account.

On the other hand, focusing on a single type of cancer and cases for which radiation treatment is exclusively recommended, along with the formation of a homogeneous cohort, made it possible to reach a better interpretation of the results. Defining waiting time is in accordance with the newly enacted legislation, which is more in keeping with the Brazilian situation. The study period covers almost 16 years, which provides a suitable interpretation of the trends of the indicators over this time.

The waiting time for radiotherapy increased over this period; this increase came at the expense of the interval between recording the diagnosis on the medical chart and the first consultation at the Specialized Unit. Therefore, efforts directed towards improving the supply of resources used for tumor staging and for the re-establishment of the clinical conditions of the patients can assist in reducing waiting times. Despite actions regarding screening the Brazilian population being at the forefront of the public health sphere, most women are already at the advanced stages when diagnosed. This study showed that treatment for those that needed it most happened more swiftly, which suggests a certain equality regarding access to radiotherapy, even though the waiting time was not ideal for all.

## Footnotes

a Department of Health. The NHS Cancer Plan: a plan for investment, a plan for reform. London (UK); 2000 [cited 2015 May 11]. Available from: http://webarchive.nationalarchives.gov.uk/+/www.dh.gov.uk/en/Publicationsandstatistics/Publications/PublicationsPolicyandGuidance/DH_4009609

b Tribunal de Contas da União, Secretaria de Fiscalização e Avaliação de Programas de Governo. Política Nacional de Atenção Oncológica. Brasília (DF); 2011. Relator: Ministro José Jorge. Available from: http://www.sbradioterapia.com.br/pdfs/relatorio-tribuna-contas-uniao.pdf

c World Health Organization, International Agency for Research on Cancer. Estimated cancer incidence, mortality and prevalence worldwide in 2012. Lyon; 2012 [cited 2014 Oct 20]. Available from: http://globocan.iarc.fr/Pages/fact_sheets_cancer.aspx

d Instituto Nacional de Câncer José Alencar Gomes da Silva, Coordenação de Prevenção e Vigilância. Estimativa 2014: incidência de câncer no Brasil. Rio de Janeiro (RJ): INCA; 2014 [cited 2015 May 7]. Available from: http://www.inca.gov.br/estimativa/2014/

e Ministério da Saúde, Secretaria de Atenção à Saúde. Portaria nº 741, de 19 de dezembro de 2005 [cited 2015 May 7]. Available from: http://www.saude.mg.gov.br/images/documentos/Portaria_741.pdf

f Brasil. Lei nº 12.732, de 22 de novembro de 2012. Dispõe sobre o primeiro tratamento de pacientes com neoplasia maligna comprovada e estabelece prazo para início. *Diario Oficial Uniao.* Nov 23, 2012; Section 1:1.

g Ministério da Saúde, Secretaria de Atenção à Saúde. Portaria nº 102, de 3 de fevereiro de 2012. Define códigos e descrição de Serviço de Radioterapia de Complexo Hospitalar e de Serviço de Oncologia Clínica de Complexo Hospitalar. Brasília (DF); 2012 [cited 2015 May 7]. Available from: http://bvsms.saude.gov.br/bvs/saudelegis/sas/2012/prt0102_03_02_2012.html

h Ministério da Saúde. DATASUS. Sistema de Informações sobre Mortalidade - SIM. Brasília (DF): Brasil. [cited 2015 Nov 5]. Available from: http://www2.datasus.gov.br/DATASUS

i Canadian Institute for Health Information. Wait time information in priority areas: definitions (updated may 2012). Ottawa; 2012 [cited 2014 Oct 20]. Available from: http://www.cihi.ca/CIHI-ext-portal/internet/EN/SubTheme/health+system+performance/access+and+wait+times/cihi010647

j Ministério da Saúde. Portaria nº 876, de 16 de maio de 2013. Dispõe sobre a aplicação da Lei nº 12.732, de 22 de novembro de 2012, que versa a respeito do primeiro tratamento com paciente com neoplasia maligna comprovada, no âmbito do Sistema Único de Saúde (SUS). *Diario Oficial Uniao.* May 17, 2013; Section 1:135-6.
