# Supplementary material for: Waiting time for radiotherapy in women with cervical cancer
Source: Rev Saude Publica. 2016 Jan 12;49:92. doi: 10.1590/S0034-8910.2015049005953 (PMC4716650; doi:10.1590/S0034-8910.2015049005953)
Supplement: Translation

# Translation: Tempo de espera para radioterapia em mulheres com câncer do colo do útero

**Authors:** Maria Isabel do Nascimento, Gulnar Azevedo e Silva

**Affiliations:** I Departamento de Epidemiologia e Bioestatística. Instituto de Saúde Coletiva. Universidade Federal Fluminense. Niterói, RJ, Brasil; II Departamento de Epidemiologia. Instituto de Medicina Social. Universidade do Estado do Rio de Janeiro. Rio de Janeiro, RJ, Brasil

**Keywords:** Neoplasias do Colo do Útero, radioterapia, Agendamento de Consultas, Listas de Espera, Referência e Consulta, Acesso aos Serviços de Saúde, Equidade no Acesso

## Abstract

**OBJETIVO:**

Descrever o tempo de espera para realização de radioterapia em casos de câncer do colo do útero.

**MÉTODOS:**

Estudo descritivo conduzido com 342 mulheres com câncer do colo do útero encaminhadas para radioterapia primária na Baixada Fluminense, RJ, período de 1995 a 2010. O tempo de espera foi calculado tendo como parâmetro o prazo de 60 dias para obter o primeiro tratamento do câncer e considerando a data da confirmação diagnóstica, a da primeira consulta oncológica e da primeira sessão de radioterapia. A comparação de medianas e proporções foi feita pelos testes de Kruskal Wallis e Qui-quadrado.

**RESULTADOS:**

A maioria das mulheres (72,2%) iniciou a radioterapia dentro de 60 dias da data da confirmação diagnóstica. A mediana do tempo de espera total foi de 41 dias. Essa mediana piorou ao longo do período e passou de 11 dias (1995 a 1996) para 64 dias (2009 a 2010). A mediana do intervalo entre confirmação diagnóstica e primeira consulta oncológica foi de 33 dias, e da primeira consulta oncológica à primeira sessão de radioterapia, foi quatro dias. Houve diferença estatisticamente significativa (p = 0,003) da mediana do tempo de espera total segundo o estadiamento do tumor, obtendo-se 56 dias, 35 dias e 30 dias para os casos classificados até IIA, IIB-IIIB, e IVA-IVB, respectivamente.

**CONCLUSÕES:**

Embora a maioria das mulheres tenha tido acesso à radioterapia dentro de 60 dias, a implementação de procedimentos para definição do estadiamento do tumor e restabelecimento das condições clínicas consumiu grande parte do tempo e precisa de aprimoramento. Ainda que o tempo de espera não tenha sido o ideal para todas as pacientes, os casos mais avançados chegaram mais rápido ao tratamento, sugerindo que o acesso à radioterapia para esse tipo de câncer tem sido alcançado com equidade.

## INTRODUÇÃO

A radioterapia contribui para a cura e tratamento paliativo de quase todos os tipos de câncer e cerca de 58,0% dos casos de câncer originados no colo do útero recebem esse tratamento.^8^ O tempo esperado para iniciar a radioterapia é considerado um indicador de qualidade do cuidado^a^ e parece influenciar os resultados do tratamento de câncer.^16^ Uma auditoria conduzida pelo Tribunal de Contas da União no Brasil mostrou cobertura de 65,9% da demanda por radioterapia em 2010 e que o tempo médio de espera pela administração da primeira sessão foi de 113,4 dias.^b^

A Agência Internacional de Pesquisa em Câncer (IARC) estimou a ocorrência de 14 milhões de casos novos de câncer no mundo em 2012.^c^ O número estimado no Brasil foi de quase 577 mil casos novos em 2014-2015, com aproximadamente 15 mil de topografia no colo do útero.^d^ Segundo a IARC, espera-se aumento da incidência de câncer do colo do útero nas próximas décadas, com projeção de ocorrência de 22.211 e 27.099 casos em 2020 e 2030, respectivamente.

No País, 45,5% dos casos de câncer do colo do útero diagnosticados entre 1995 e 2002 encontravam-se em estádios avançados,^14^ aumentando a demanda por serviços de atenção oncológica que ofertavam radioterapia e exigindo melhor resolubilidade desses serviços, uma vez que se tratam de casos cuja oportunidade de tratamento curativo se centrava na radioterapia. Estima-se que para cada mil casos novos de câncer, 500 a 600 necessitem cirurgia oncológica, 700, de quimioterapia e 600, da radioterapia como parte do esquema terapêutico.^e^ A demanda para câncer do colo do útero pode ser maior, uma vez que a radioterapia foi indicada para 69,8% das pacientes com doença nos estádios II e III do Instituto Nacional de Câncer (INCA), entre 1999 e 2004.^6^

Há grande preocupação em melhorar o acesso ao tratamento do câncer no Brasil, com medidas direcionadas para a organização da rede de serviços e maior disponibilização de recursos tecnológicos. Em 2012, foi promulgada a lei que regulamenta a tempestividade aceitável de até 60 dias para se estabelecer o primeiro tratamento de novos casos de câncer.^f^ Tão importante quanto a legislação é o diagnóstico da situação para entender as causas de demora e planejar melhor a abordagem da doença. É oportuno conhecer: (i) a extensão do tempo de espera total e dos seus intervalos componentes impostos às mulheres até iniciar a radioterapia para câncer do colo do útero; (ii) se algum componente tem maior impacto no tempo esperado para iniciar a radioterapia; e (iii) se o tempo de espera para tumores encaminhados para radioterapia potencialmente curativa e paliativa é o mesmo.

O objetivo do estudo foi descrever o tempo de espera para realização de radioterapia em casos de câncer do colo do útero.

## MÉTODOS

Este estudo descritivo apresenta dados de uma coorte de mulheres com câncer do colo do útero, identificadas entre outubro de 1995 e agosto de 2010 nos registros dos setores de anatomia patológica ou patologia cervical de um hospital escola (Hospital Geral de Nova Iguaçu – HGNI). O HGNI integra a rede do Sistema Único de Saúde (SUS) e está localizado na Baixada Fluminense, parte da Região Metropolitana I do Estado do Rio de Janeiro. O hospital abriga um polo para colposcopia com habilitação para esclarecimento e tratamento de lesões precursoras de câncer do trato genital inferior e para diagnóstico do câncer do colo do útero. Após o diagnóstico e os cuidados iniciais, as pacientes com doença invasora cervical são encaminhadas para tratamento em unidades públicas especializadas da cidade do Rio de Janeiro ou para o Serviço Isolado, credenciado pelo SUS, para Radioterapia e Quimioterapia, na Baixada Fluminense.

Foram identificados 667 casos novos de câncer do colo do útero livres de tratamento no momento diagnóstico no HGNI, no período. Entre eles, 51 evoluíram para óbito antes de iniciar o tratamento do câncer, 109 foram eleitos para tratamento cirúrgico primário e para 26 não se dispunha de informações suficientes que permitissem a identificação no seguimento. Assim, 481 pacientes com indicação de radioterapia exclusiva eram elegíveis para o estudo. Destes, foram excluídos 128 casos com tratamento apenas em unidades oncológicas da capital, onde se conta com rede muito mais abrangente de estabelecimentos habilitados pelo SUS para radioterapia^g^ em comparação ao único serviço isolado em funcionamento na Baixada Fluminense. Dos 353 casos, oito foram excluídos por recusarem o tratamento recomendado e três por, embora encaminhados, terem a radioterapia contraindicada. A população estudada foi composta por 342 mulheres submetidas à radioterapia no Serviço Isolado de Radioterapia e Quimioterapia localizado na Baixada Fluminense.

A média de idade das pacientes foi de 51,7 anos (DP: 13,8 anos; variação: 25 a 90 anos). A análise morfológica mostrou maior frequência de tumores do tipo escamoso (90,9%), seguida de adenocarcinoma (5,8%), adenoescamoso (1,5%) e outros tipos (1,8%). O estadiamento clínico mostrou que grande parte dos casos apresentava extensão de doença para além da estrutura cervical (90,4%).

As informações analisadas foram obtidas dos prontuários de pacientes atendidas no HGNI e no Serviço Isolado, bem como dos arquivos eletrônicos do Registro Hospitalar de Câncer instalado no Serviço Isolado. Também foi consultada a base de óbitos do Sistema de Informação sobre Mortalidade^h^ do Estado do Rio de Janeiro para complementação de informações. Foram coletados dados demográficos, relacionados ao tumor, sobre condições de encaminhamento e de tratamento. Foram verificadas, sobretudo, a extensão do tempo de espera total e seus intervalos componentes, cujos cálculos foram efetuados da seguinte forma:

- Intervalo entre a confirmação diagnóstica e o início da radioterapia (tempo de espera total): tempo (em dias) transcorrido entre a data do registro do diagnóstico histopatológico no prontuário da paciente no HGNI e a data da primeira sessão de radioterapia no Serviço Isolado.
- Intervalo entre a confirmação diagnóstica e a primeira consulta oncológica no Serviço Isolado: tempo (em dias) transcorrido entre a data do registro do diagnóstico histopatológico no prontuário da paciente no HGNI e a data da primeira consulta oncológica no Serviço Isolado.
- Intervalo entre a primeira consulta oncológica no Serviço Isolado e a primeira sessão de radioterapia: tempo (em dias) transcorrido entre a data da primeira consulta oncológica no Serviço Isolado e a data da primeira sessão de radioterapia.

A análise do tempo de espera total foi feita segundo as seguintes categorias: ≤ 15 dias *versus* > 15 dias; ≤ 30 dias *versus* > 30 dias; ≤ 60 dias *versus* > 60 dias; ≤ 90 dias *versus* > 90 dias; ≤ 120 dias *versus* > 120 dias.

As demais variáveis analisadas foram: idade ao diagnóstico (≥ 50 anos *versus* < 50 anos), estadiamento clínico (tumores até IIA; tumores IIB-IIIB; tumores IVA-IVB), estabelecido segundo critérios da Federação Internacional de Ginecologia e Obstetrícia (FIGO).^2^

A análise incluiu estimativa de média, desvio padrão (DP), mediana, intervalo interquartil (IRQ), frequências absolutas e relativas. Foram aplicados testes não paramétricos para comparar as variáveis contínuas (teste Kruskal Wallis) e variáveis categóricas (teste Qui-quadrado), considerando nível de significância de 5%.

O estudo foi aprovado pelo Comitê de Ética em Pesquisa do HGNI (Processo 259.780, de 26 de março de 2013).

## RESULTADOS

Todas as 342 mulheres receberam tratamento por radioterapia externa isolada ou combinada a terapias complementares, no Serviço Isolado de Radioterapia e Quimioterapia, resultando em oito diferentes esquemas de tratamento (Tabela 1).

**Tabela 1:** Tempo de espera para radioterapia no câncer do colo do útero: distribuição das características selecionadas de 342 casos tratados. Baixada Fluminense, RJ, 1995 a 2010.

Variável	n	%
Faixa etária (anos)		
20 a 29	14	4,1
30 a 39	51	14,9
40 a 49	100	29,2
50 a 59	87	25,4
60 a 69	45	13,2
70 a 79	36	10,5
80 e mais	9	2,6
Estadiamento clínico		
IA a IB	33	9,6
IIA a IIB	135	39,5
IIIA a IIIB	157	45,9
IVA a IVB	17	5,0
Morfologia		
Escamoso	311	90,9
Adenocarcinoma	20	5,8
Adenoescamoso	5	1,5
Outros	6	1,8
Esquema de tratamento com Radioterapia Externa +		
Braquiterapia e quimioterapia	60	17,5
Braquiterapia	94	27,5
Reforço	20	5,8
Quimioterapia	58	17,0
Quimioterapia e reforço	31	9,1
Braquiterapia e reforço	1	0,3
Braquiterapia e reforço e quimioterapia	1	0,3
Radioterapia externa isolada	77	22,5

A espera pela radioterapia, avaliada pela mediana do intervalo entre a confirmação diagnóstica e a primeira sessão de radioterapia (tempo de espera total) ao longo do período (1995 a 2010), foi de 41 dias (IRQ: 50 dias). A variação foi de quase seis vezes, passando de 11 dias no biênio inicial (1995 a 1996) para 64 dias no biênio final (2009 a 2010). A mediana do intervalo entre a confirmação diagnóstica e a primeira consulta oncológica no Serviço Isolado e da primeira consulta oncológica à primeira sessão de radioterapia foi de 33 dias (IRQ: 50 dias) e quatro dias (IRQ: sete dias), respectivamente. Com exceção do biênio 1995-1996, para o intervalo entre a primeira consulta oncológica e o início da radioterapia, a extensão das médias dos componentes de tempo analisados foi sistematicamente maior que a extensão das medianas, ao longo de todos os períodos. Esse achado apontou para a presença de valores extremos à direita, representativos de casos que, embora tenham tido acesso à radioterapia, isso ocorreu, para alguns, de modo muito postergado (Tabela 2).

**Tabela 2:** Tempo de espera para radioterapia no câncer do colo do útero: média, mediana e intervalo interquartil em dias da espera total e dos intervalos componentes por biênios. Baixada Fluminense, RJ, 1995 a 2010.

Biênio	Intervalo total entre confirmação diagnóstica e início da radioterapia no serviço isolado			Intervalo entre confirmação diagnóstica e primeira consulta oncológica no serviço isolado			Intervalo entre primeira consulta oncológica e início da radioterapia no serviço isolado		

	Média	Mediana	IRQ*	Média	Mediana	IRQ	Média	Mediana	IRQ
1995 a 1996	21,6	11	12	17,1	5	2	4,5	5	5
1996 a 1997	15,1	11	15	8,5	6	6	6,5	3	5
1999 a 2000	35,3	15	40	30,6	12	35	4,6	1	1
2001 a 2002	29,9	17	26	21,5	7	20	8,3	6	8
2003 a 2004	40,6	30	39,5	30,1	16,5	38	10,4	5	9,5
2005 a 2006	77,1	58	39	73,2	56,5	35	3,9	1	5,5
2007 a 2008	69,1	60	37	62,9	56	31	6,2	4	7
2009 a 2010	82,1	64	26	71,2	53	22	10,9	8,5	8
1995 a 2010	48,3	41	50	41,1	33	50	7,2	4	7

* IRQ (intervalo interquartil).

A análise do estadiamento clínico mostrou que 16,1% das mulheres encontravam-se em estádios mais precoces (até IIA); 78,9% foram classificadas em estágios intermediários (IIB-IIIB) e 5,0% tinham doença mais avançada (estágios IVA-IVB). Tais condições mostraram relação estatisticamente significativa (p = 0,003) com o tempo de espera total, obtendo-se medianas de 56 dias, 35 dias e 30 dias para os casos classificados nos grupos até IIA, de IIB a IIIB, e IVA-IVB, respectivamente.

A representação esquemática dos diferentes componentes do tempo de espera mostrou que o aumento do intervalo entre a confirmação diagnóstica e a primeira sessão de radioterapia foi mais discreto entre o biênio 1997 a 1998 e 1999 a 2000, deteriorando mais acentuadamente a partir de 2001 e 2002. Durante todo o período, o intervalo entre a primeira consulta oncológica e o início da radioterapia manteve-se constante, não ultrapassando 10 dias (Figura).

**Figura:** Tempo de espera para radioterapia no câncer do colo do útero: avaliação da mediana do tempo de espera total e dos intervalos componentes ao longo dos biênios do estudo. Baixada Fluminense, RJ, 1995 a 2010.

Em relação ao tempo de espera total, a proporção de casos com acesso ao tratamento foi de 30,4%, 43,8%, 72,2%, 88,3% e 94,1% dentro de até 15 dias, 30 dias, 60 dias, 90 dias e até 120 dias, respectivamente. Até finalizar os três primeiros biênios, mais da metade dos casos iniciaram a radioterapia com até 15 dias. Essa proporção caiu de 44,9% (2001 a 2002) para zero (2005 a 2006 e 2009 a 2010) (Tabela 3).

**Tabela 3:** Tempo de espera total a partir da confirmação diagnóstica até o início da radioterapia no câncer do colo do útero: proporção de casos com acesso ao tratamento em 15 dias, 30 dias, 60 dias, 90 dias e 120 dias. Baixada Fluminense, RJ, 1995 a 2010. (N = 342)

Variável	Total		Acesso em até 15 dias		Acesso em até 30 dias		Acesso em até 60 dias		Acesso em até 90 dias		Acesso em até 120 dias	

	n	%	n	%	n	%	n	%	n	%	n	%
Total			104	30,4	150	43,8	247	72,2	302	88,3	322	94,1
Biênio												
1995 a 1996	10	2,9	7	70,0	9	90,0	9	90,0	9	90,0	10	100
1997 a 1998	47	13,7	34	72,3	41	87,2	46	97,9	47	100	47	100
1999 a 2000	43	12,6	22	51,2	29	67,4	34	79,1	37	86,0	41	95,4
2001 a 2002	49	14,3	22	44,9	34	69,4	43	87,8	47	96,0	47	96,0
2003 a 2004	56	16,4	17	16,3	29	51,8	48	85,7	50	89,3	53	94,6
2005 a 2006	48	14,0	0	0	3	6,2	26	54,2	39	81,2	43	89,6
2007 a 2008	51	14,9	2	1,9	5	9,8	26	51,0	41	80,4	47	92,2
2009 a 2010	38	11,1	0	0	0	0	15	39,5	32	84,2	34	89,5
p				< 0,001		< 0,001		< 0,001		0,032		0,364
Estadiamento												
De IA a IIA	55	16,1	10	18,2	15	27,3	35	63,6	43	78,2	47	85,5
De IIB a IIIB	270	78,9	87	32,2	126	46,7	201	74,4	244	90,4	260	96,3
De IVA e IVB	17	5,0	7	41,2	9	52,9	11	64,7	15	88,2	15	88,2
p				0,073		0,023		0,206		0,037		0,004
Faixa etária (anos)												
≥ 50	177	51,7	57	32,2	82	46,3	128	72,3	155	87,6	165	93,2
< 50	165	48,3	47	28,4	68	41,2	119	72,1	147	90,0	157	95,1
p				0,455		0,341		0,968		0,662		0,447

O acesso à radioterapia com até 60 dias foi alcançado por proporção relativamente alta de mulheres (72,2%), considerando todo o período. A proporção atingiu a casa dos 90,0% nos dois primeiros biênios. Entretanto, o acesso à radioterapia até 60 dias veio diminuindo gradativamente de 2001 a 2002 (87,3%) até 2009 a 2010, quando 39,5% das pacientes com câncer do colo do útero tinham iniciado a radioterapia, dentro do referido prazo.

Independentemente do ponto de corte definidor dos intervalos (15, 30, 60, 90 e 120 dias), a proporção de mulheres com doença nos estádios IIB-IIIB e IVA-IVB que chegaram mais rápido à radioterapia foi maior do que aquelas classificadas nos estádios mais precoces até IIA.

O acesso à radioterapia para mulheres com menos de 50 anos foi semelhante ao daquelas com 50 anos ou mais, em todos os pontos de corte de espera analisados.

## DISCUSSÃO

Este estudo fornece informações inéditas sobre o tempo esperado pelas mulheres diagnosticadas com câncer do colo do útero em hospital de ensino até iniciar a radioterapia em Serviço Isolado. Grande parte (72,2%) das mulheres com a doença teve acesso à radioterapia dentro do prazo de 60 dias a contar da confirmação diagnóstica, registrada no prontuário. Ainda que a mediana do tempo de espera tenha sido de 41 dias, seus intervalos componentes influenciaram de modo diferente a espera pela radioterapia. Enquanto o intervalo entre a confirmação diagnóstica e a primeira consulta oncológica no serviço isolado aumentou de cinco para 53 dias do biênio inicial (1995 a 1996) para o final (2009 a 2010), o intervalo entre a primeira consulta oncológica e a efetiva administração do tratamento manteve-se estável com menos de 10 dias ao longo de todo o período.

Estudos sobre tempo de espera para tratamento de câncer são escassos em todo o mundo e diferem nas definições dos intervalos utilizados e tipos de tumores e de terapêutica administrada, dificultando a comparabilidade. Um dos poucos estudos no tema desenvolvidos no Brasil teve a data da primeira consulta no Departamento de Cirurgia de Cabeça e Pescoço do Hospital de Heliópolis, São Paulo, SP, como referência inicial.^1^ A avaliação contou com 217 pacientes com câncer de laringe diagnosticados entre 1996 e 2004 e a mediana para o início da radioterapia foi de 58 dias.

Em Ontário, Canadá, obteve-se mediana de 5,7 semanas da cirurgia ou biópsia até a primeira sessão de radioterapia no câncer do colo do útero diagnosticado em 2001 e 2002.^3^ Naquele país, a partir de 2011, o *Canadian Institute for Health Information* estabeleceu como padrão o prazo de 28 dias para o tratamento do câncer por radioterapia.^i^ A definição considera o tempo de espera como o número de dias transcorridos entre a data que o paciente é considerado pronto para tratar e a data da primeira sessão de radioterapia. A instituição assinalou que o tempo despendido para o plano terapêutico oncológico faz parte do tempo de espera, mas não incluiu o tempo necessário para o estadiamento clínico do tumor. Casos do mesmo tipo diagnosticados mais recentemente (2003 a 2007) em Auckland, Nova Zelândia, tiveram mediana de 26 dias contados da data de decisão de tratar até a administração da terapia ionizante.^5^

No Reino Unido, estudos que sucederam a implantação do *NHS Cancer Plan* ^a^ forneceram informações sobre o tempo de espera para radioterapia e indicaram possíveis causas relacionadas ao prolongamento dos intervalos. A partir de dados de 7.051 casos de câncer do útero, para os quais os autores não especificaram a localização, a mediana para iniciar a radioterapia foi de 68 dias a contar da data de confirmação diagnóstica e 42,3% dos casos receberam a primeira sessão de radioterapia dentro de 60 dias.^12^ Como fatores explicativos para o tempo esperado para o tratamento de qualquer tipo de câncer, os autores sugerem falta de equipe treinada e de equipamentos, mudanças no manejo das doenças malignas que estão levando à maior demanda por radioterapia e aumento da carga de trabalho devido à incidência crescente de todos os tipos de câncer combinados, embora se verifique redução nos casos novos de câncer do colo do útero.^15^ Coles et al^7^ avaliaram o intervalo entre a primeira consulta oncológica e a primeira sessão de radioterapia no *Addenbrooke’s Hospital*, Cambridge, UK, em um grupo de pacientes com câncer do colo do útero. Os autores detectaram aumento da mediana de 14 dias (1996) para 18 dias (1998) e depois para 35 dias (2001). A opção por regimes de radioterapia mais fracionados, de maior complexidade, acompanhado de falta de aceleradores lineares e de equipe treinada pode explicar parcialmente tais achados.

Assim, a análise crítica da situação pode ajudar no direcionamento das ações para encurtar o tempo de espera para o tratamento do câncer. Os atuais resultados indicam que o intervalo entre a confirmação diagnóstica e a primeira consulta oncológica consumiu a maior parte do tempo que antecedeu a radioterapia. Esse intervalo comporta a implementação de medidas com diferentes graus de complexidade não só para o estabelecimento do estadiamento do tumor, mas principalmente para a manutenção da vida das pacientes que chegam em condições clínicas precárias no momento do diagnóstico.

A linha de cuidado do câncer do colo do útero percorre inúmeros serviços e depende da disponibilidade de diferentes tecnologias,^4^ as quais são imprescindíveis para a definição do estadiamento e para a recuperação clínica das pacientes. Nessa fase, procedimentos de alta complexidade como hemotransfusão, hemodiálise e cirurgias de médio e grande porte, de acesso ainda restrito em algumas regiões do País, são frequentemente requisitados. Equipes multiprofissionais devidamente habilitadas para lidar com câncer e questões logísticas que parecem simples, como a disponibilidade de transporte, mas que nem sempre são de acesso fácil para pacientes e profissionais de saúde também contribuem para o tempo de espera.

Desde 2012, o problema do tempo esperado para iniciar o tratamento do câncer tem merecido atenção especial das autoridades governamentais. Depois de sancionada a lei^f^ que versa sobre o primeiro tratamento de pacientes com câncer, seguiu-se a Portaria^j^ que dispõe sobre a aplicação da Lei, que fixa o prazo de 60 dias para iniciar efetivamente o tratamento de neoplasias malignas no SUS. Isso direciona a questão para o lado judicial, e não como um problema tratado cientificamente no primeiro mundo. O *European Cancer Health Indicador Project* (EUROCHIP)^9^ objetiva analisar as desigualdades em câncer, baseando-se em dados de registros de câncer de base populacional e em indicadores de estadiamento, demora do tratamento e adesão a protocolos clínicos. O citado projeto definiu que a demora para o tratamento do câncer seria um indicador importante relacionado ao sistema de saúde a ser analisado. A avaliação feita pelo EUROCHIP 3^13^ concluiu que 37,0% dos 86 registros de câncer de base populacional da Europa dispunham de dados sobre a demora para tratamento, sugerindo que se trata de questão crítica que merece mais atenção de todos os setores envolvidos. Os autores destacaram a importância da análise de situação como uma maneira de melhorar a compreensão das causas da espera. Para eles, a demora pode decorrer não só da agenda para cirurgia, quimioterapia e radioterapia, mas principalmente de falta de adequabilidade dos recursos disponíveis, da educação do paciente, do padrão de encaminhamento e da espera longa para consulta com especialistas e para exames de imagem essenciais ao diagnóstico.

No presente estudo, quase 86,0% dos casos encontravam-se nos estádios II ou III no momento do diagnóstico, proporção superior à relatada em análises conduzidas na cidade de Vitória^10^ (75,4%) e no Rio de Janeiro^6^ (68,3%). Pelo EUROCHIP 3,^13^ o estádio ao diagnóstico sinaliza a demora que os pacientes enfrentam até obter a confirmação do diagnóstico do câncer. Para estes autores, o estádio ao diagnóstico reflete fatores como conhecimento da população sobre sinais de câncer, acessibilidade aos hospitais e centros diagnósticos e habilidade de profissionais para lidar com o câncer. Para um tipo de câncer considerado totalmente evitável, como é caso do câncer do colo do útero, mas ainda detectado em fase muito avançada, a veiculação de informações instrumentalizando a população sobre os sinais e sintomas da doença, a disponibilidade de profissionais e de exames na atenção básica e a importância da adesão ao rastreamento pode otimizar os recursos e captar casos em estádios mais precoces. Isso pode reduzir a demanda por radioterapia. Além disso, parte das mulheres que chegaram com estadiamento avançado não foram submetidas a rastreamento, sugerindo que os programas de rastreamento não foram efetivos para captar todas as mulheres das faixas etárias-alvo em algumas áreas.

Questão levantada no presente estudo foi se o tempo de espera para tratamento de tumores, encaminhados para radioterapia potencialmente curativa e para radioterapia paliativa, seria o mesmo. Enquanto as pacientes com doença em estádios mais precoces, classificados em até IIA, iniciaram o tratamento com mediana de 56 dias, aquelas em situações mais avançadas chegaram ao tratamento dentro de 35 dias (tumores IIB até IIIB) ou de 30 dias de mediana (tumores IVA/IVB). Estudo de base populacional desenvolvido na Polônia, restrito a pacientes com câncer de mama, encontrou média de tempo de espera para iniciar o tratamento maior nos casos de doença localizada.^11^ Os autores argumentaram que o diagnóstico de doença mais avançada é mais rápido porque requer uma série mais curta de procedimentos, de menor custo, com maior facilidade de realização e de interpretação, comparando com os tumores em estádios mais precoces. Os atuais resultados sugerem que no caso do câncer do colo do útero, a extensão do tempo de espera foi provavelmente influenciada por decisões clínicas tomadas para agilizar o tratamento dos casos mais graves.

Este estudo tem algumas limitações. Foi desenvolvido com dados retrospectivos, registrados com finalidade basicamente assistencial e administrativa. Entretanto, a coleta cuidadosa dos dados com busca incessante aos marcos temporais, aqui utilizados, contribuiu para minimizar as possíveis inconsistências. Embora o tempo de espera para iniciar a radioterapia seja importante indicador para o tratamento do câncer, o estudo se manteve no plano quantitativo e não avaliou as condições técnicas de implementação da radioterapia e a completude do regime programado. O expressivo número de casos com tratamento apenas em unidades oncológicas da capital motivou a exclusão de mais de 1/4 das pacientes com indicação de radioterapia. Ainda que o impacto de tal decisão seja inegável, os resultados alinham-se aos propósitos do estudo e expressam um indicador da atenção oncológica ofertada na Baixada Fluminense, região que conta com um único serviço habilitado pelo SUS para radioterapia. Diante do panorama atual de grande interesse das autoridades governamentais para o câncer, avaliações da implementação da braquiterapia, que não foi feita aqui, poderão trazer importantes contribuições na abordagem do câncer do colo do útero, principalmente se o intervalo contado a partir do término da irradiação externa for devidamente contemplado.

Por outro lado, o foco em um tipo único de câncer indicado para tratamento radioterápico exclusivo, com formação de coorte homogênea, permitiu melhor interpretação dos resultados. A definição do tempo de espera está de acordo com a legislação recém promulgada, conferindo maior sintonia com o cenário brasileiro. O período do estudo estendeu-se por quase 16 anos, fornecendo interpretação adequada de tendência dos indicadores ao longo do tempo.

O tempo de espera para radioterapia aumentou ao longo do período e o incremento deu-se à custa do intervalo entre o registro do diagnóstico no prontuário e a primeira consulta no Serviço Isolado. Nesse contexto, os esforços direcionados ao suprimento de recursos usados para o estadiamento tumoral e para o restabelecimento das condições clínicas das pacientes podem auxiliar na redução do tempo de espera. Em que pese as ações de rastreamento populacional em voga no Brasil para o âmbito da saúde pública, a maioria das mulheres chega ao diagnóstico em estádio avançado de doença. Este estudo mostrou que o tratamento das que mais dele necessitavam começou mais rápido, sugerindo acesso à radioterapia com equidade, ainda que o tempo de espera não tenha sido o ideal para todas.

## Footnotes

a Department of Health. The NHS Cancer Plan: a plan for investment, a plan for reform. London (UK); 2000 [citado 2015 mai 11]. Disponível em: http://webarchive.nationalarchives.gov.uk/+/www.dh.gov.uk/en/Publicationsandstatistics/Publications/PublicationsPolicyandGuidance/DH_4009609

b Tribunal de Contas da União, Secretaria de Fiscalização e Avaliação de Programas de Governo. Política Nacional de Atenção Oncológica. Brasília (DF); 2011. Relator: Ministro José Jorge. Disponível em: http://www.sbradioterapia.com.br/pdfs/relatorio-tribuna-contas-uniao.pdf

c World Health Organization, International Agency for Research on Cancer. Estimated cancer incidence, mortality and prevalence worldwide in 2012. Lyon; 2012 [citado 2014 out 20]. Disponível em: http://globocan.iarc.fr/Pages/fact_sheets_cancer.aspx

d Instituto Nacional de Câncer José Alencar Gomes da Silva, Coordenação de Prevenção e Vigilância. Estimativa 2014: incidência de câncer no Brasil. Rio de Janeiro (RJ): INCA; 2014 [citado 2015 mai 7]. Disponível em: http://www.inca.gov.br/estimativa/2014/

e Ministério da Saúde, Secretaria de Atenção à Saúde. Portaria nº 741, de 19 de dezembro de 2005 [citado 2015 mai 7]. Disponível em: http://www.saude.mg.gov.br/images/documentos/Portaria_741.pdf

f Brasil. Lei nº 12.732, de 22 de novembro de 2012. Dispõe sobre o primeiro tratamento de pacientes com neoplasia maligna comprovada e estabelece prazo para início. *Diario Oficial Uniao*. 23 nov 2012; Seção 1:1.

g Ministério da Saúde, Secretaria de Atenção à Saúde. Portaria nº 102, de 3 de fevereiro de 2012. Define códigos e descrição de Serviço de Radioterapia de Complexo Hospitalar e de Serviço de Oncologia Clínica de Complexo Hospitalar. Brasília (DF); 2012 [citado 2015 mai 7]. Disponível em: http://bvsms.saude.gov.br/bvs/saudelegis/sas/2012/prt0102_03_02_2012.html

h Ministério da Saúde. DATASUS. Sistema de Informações sobre Mortalidade - SIM. Brasília (DF): Brasil [citado 2015 nov 5]. Disponível em: http://www2.datasus.gov.br/DATASUS

i Canadian Institute for Health Information. Wait time information in priority areas: definitions (updated may 2012). Ottawa; 2012 [citado 2014 out 20]. Disponível em: http://www.cihi.ca/CIHI-ext-portal/internet/EN/SubTheme/health+system+performance/access+and+wait+times/cihi010647

j Ministério da Saúde. Portaria nº 876, de 16 de maio de 2013. Dispõe sobre a aplicação da Lei nº 12.732, de 22 de novembro de 2012, que versa a respeito do primeiro tratamento com paciente com neoplasia maligna comprovada, no âmbito do Sistema Único de Saúde (SUS). *Diario Oficial Uniao*. 17 mai 2013; Seção 1:135-6.

## DESTAQUES

O estudo analisou o tempo esperado para obter o primeiro tratamento por radioterapia em mulheres com câncer do colo do útero, com diagnóstico nos anos de 1995 até 2010, em um estabelecimento de saúde do Sistema Único de Saúde (SUS) localizado na Baixada Fluminense.

Os resultados mostraram que 72,2% das mulheres obtiveram a radioterapia dentro de 60 dias, contados da data da confirmação diagnóstica até a primeira sessão de tratamento (tempo de espera total). A análise por biênios mostrou que a mediana do tempo de espera total piorou ao longo do tempo, passando de 11 dias (1995-1996) para 64 dias (2009-2010). Desmembrando esse tempo total, o intervalo entre a confirmação diagnóstica e a primeira consulta oncológica (mediana de 33 dias) consumiu a maior parte do tempo (variação de cinco dias no biênio 1995-1996 a 56,5 dias no biênio 2005-2006) comparando com o intervalo entre a primeira consulta oncológica e a primeira sessão de radioterapia, que teve mediana de quatro dias e variação de 1 (biênios 1999-2000 e 2005-2006) a 8,5 dias (biênio 2009-2010).

Ações que visem o encurtamento do intervalo entre a confirmação diagnóstica e a primeira consulta oncológica, fase do estabelecimento do estadiamento do tumor e definição da conduta terapêutica, devem ser priorizadas.

Rita de Cássia Barradas Barata

Editora Científica
